# New role of astrocytes in neuroprotective mechanisms after ischemic stroke

**DOI:** 10.1055/s-0043-1770352

**Published:** 2023-08-30

**Authors:** Xiaoyun Xie, Jingli Liu

**Affiliations:** 1 Guangxi Medical University, The First Affiliated Hospital, Department of Neurology, Nanning, Guangxi, China.

**Keywords:** Astrocytes, Ischemic Stroke, Neurogenesis, Phagocytosis, Ischemic Preconditioning, Neuroprotective Agents, Astrócitos, AVC Isquêmico, Neurogênese, Fagocitose, Precondicionamento Isquêmico, Fármacos Neuroprotetores

## Abstract

Astrocytes are the most abundant cell subtypes in the central nervous system. Previous studies believed that astrocytes are supporting cells in the brain, which only provide nutrients for neurons. However, recent studies have found that astrocytes have more crucial and complex functions in the brain, such as neurogenesis, phagocytosis, and ischemic tolerance. After an ischemic stroke, the activated astrocytes can exert neuroprotective or neurotoxic effects through a variety of pathways. In this review, we will discuss the neuroprotective mechanisms of astrocytes in cerebral ischemia, and mainly focus on reactive astrocytosis or glial scar, neurogenesis, phagocytosis, and cerebral ischemic tolerance, for providing new strategies for the clinical treatment of stroke.

## Resumo

Os astrócitos são os subtipos de células mais abundantes no sistema nervoso central. Estudos anteriores acreditavam que os astrócitos são células de suporte no cérebro, que apenas fornecem nutrientes para os neurônios. No entanto, estudos recentes descobriram que os astrócitos têm funções mais cruciais e complexas no cérebro, como neurogênese, fagocitose e tolerância isquêmica. Após um acidente vascular cerebral isquêmico, os astrócitos ativados podem exercer efeitos neuroprotetores ou neurotóxicos através de uma variedade de vias. Nesta revisão, discutiremos os mecanismos neuroprotetores dos astrócitos na isquemia cerebral, e focaremos principalmente na astrocitose reativa ou cicatriz glial, neurogênese, fagocitose e tolerância isquêmica cerebral, para fornecer novas estratégias para o tratamento clínico do acidente vascular cerebral.

## INTRODUCTION

Ischemic stroke is one of the leading causes of disability and death in adults worldwide. 1 Currently, intravenous thrombolysis and intravascular thrombectomy are effective treatments for ischemic stroke. 2 However, the strict therapeutic time window limits their clinical application, and many patients die or become disabled due to lack of timely therapy. Therefore, it is necessary to explore effective treatment strategies for ischemic stroke outside the therapeutic time window. Excitatory amino acid toxicity, oxidative stress, calcium overload, and inflammatory response caused by cerebral ischemia can seriously impair the functions of neurons, glial cells, and endothelial cells, which lead to platelet activation, glial hyperplasia, immune cells activation, and death of neurocytes. 3 4

Although cerebral ischemia affects all cell components in the brain, including neurons, glial cells, endothelial cells etc., most studies often focus on the protection of neurons and fail to become effective clinical treatments. 5 6 Merely protecting damaged neurons may not be enough to find effective treatment strategies. Therefore, it is necessary to consider therapeutic approaches that benefit multiple cell types.

Astrocytes are the most abundant cell type in the brain, accounting for about 40% of all brain cells. 7 According to the morphology and tissue locations, brain astrocytes mainly include the following types: radial astrocytes around the ventricle, fibrous astrocytes in white matter, protoplasm astrocytes in gray matter, and velate astrocytes in cerebellar granular layer, among others. 7 8

**Figure 1:**
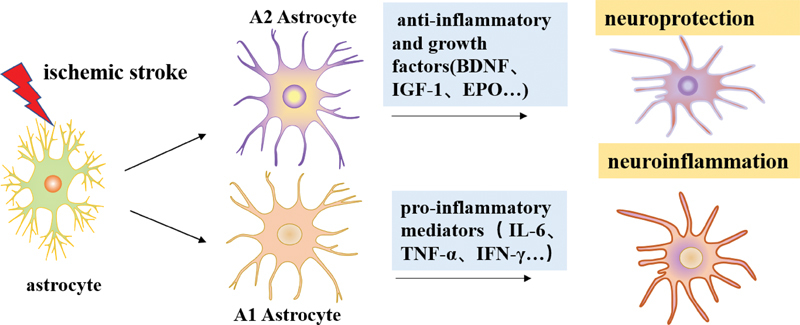
Diagram showing the pro-inflammatory and anti-inflammatory reactive astrocytes in the ischemic brain. After ischemic stroke, astrocytes become activated and polarized into pro-inflammatory A1 and anti-inflammatory A2 phenotypes. **Abbreviations:** BDNF, brain derived neurotrophic factor; IGF-1, insulin-like growth factor 1; EPO, hemopoietin; IL-6, interleukin-6; TNF-α, tumor necrosis factor–α; IFN-γ, interferon-gamma.

Under physiological conditions, astrocytes have a variety of functions such as metabolic support, nutrition, as well as regulating neurotransmitters, participating in the formation of the blood-brain barrier, regulating synapsis, and promoting neurogenesis. 9 10 11 Astrocytes are activated rapidly and have a dual role in cerebral ischemia, showing two different functional phenotypes, namely, the neurotoxic type A1 astrocytes mainly induced by inflammation and the neuroprotective A2 type reactive astrocytes induced by ischemia ( Figure 1 ). 12 13 These cells play essential roles in the brain and may become novel therapeutic targets of ischemic stroke. Consequently, we will review the endogenous neuroprotective mechanisms of astrocytes after an ischemic stroke, and introduce reactive astrocytosis or glial scar, neurogenesis, phagocytosis, and cerebral ischemia tolerance.

## REACTIVE ASTROCYTES AND ISCHEMIC STROKE

When an ischemic stroke occurs, the morphology, function, and molecular expression profile of astrocytes change significantly. Within minutes of brain ischemia, cytokines produced by damaged neurons, as well as glial cells in the core area of the infarct and the penumbra, trigger astrocyte activation. 8 This is also known as reactive astrogliosis, and it is characterized by cell hypertrophy, proliferation, and increased expression of glial fibrillary acidic protein (GFAP), changing the expression of many molecules involved in cell structure, energy metabolism, gene transcription, intracellular signal transduction, and membrane transport proteins. 14 15

Within a few days of ischemia, the reactive astrocytes form glial scars around the ischemic lesion. Glial scars can isolate the injured site and prevent the expansion of inflammation, but the astrocytes in the scars also release related molecules that inhibit axon regeneration. 16 17 Furthermore, the activated astrocytes can mediate neuroinflammation by releasing pro- or anti-inflammatory factors, which may subsequently play a neurotoxic or neuroprotective role in ischemic strokes.

Additionally, during the later stages of ischemic stroke, activated astrocytes release extracellular matrix molecules such as thrombospondin, 18 hevin, 19 and secreted protein acidic rich in cysteine (SPARC), 20 which may induce synaptic structure and function, thereby protecting the brain from synaptic damage caused by ischemia. Therefore, induction of the different astrocyte phenotypes in a controlled manner may be essential for the development of new methods to limit harmful neuroinflammation and promote neuroprotection or neurorestoration after ischemic stroke.

## REACTIVE ASTROCYTOSIS AND GLIAL SCAR AFTER ISCHEMIC STROKE

During acute ischemic strokes, in addition to restricting the lesion to minimize the area of inflammation, reactive astrocytes may also limit the secretion of diffusion factors from the injured area into remote region. 21 Depending upon the degree of injury, mild astrogliosis can disappear over time, while in more severe injuries the glial scar formation can be permanent. 14 22 This is consistent with astrocytosis in the chronic infarct lesions of stroke patients. 23

Compared to normal astrocytes, those devoid of GFAP and vimentin (GFAP ^−/−^ Vim ^−/−^ ), as well as those exposed to oxygen-glucose deprivation/reperfusion (OGD/R), have a reduced ability to scavenge reactive oxygen species and increased cell death, indicating that the astrocyte's intermediate filament system plays an important role in oxidative stress. 24 It has been reported that GFAP and scar formation were significantly lessened in the CD36 (a class B scavenger receptor) knockout (KO) mice after cerebral ischemia, suggesting that targeting CD36 may offer effective strategies for reducing glial scar formation in ischemic strokes. 25 Consistently, GFAP ^−/−^ Vim ^−/−^ mice reduce glial hyperplasia and scar formation, with longer posttraumatic healing time and more significant synaptic loss. 26 Traditionally, astrocytes in glial scars secrete a large amount of growth-inhibiting extracellular matrix dominated by chondroitin sulfate proteoglycans (CSPG), which forms a physical barrier to inhibit axon regeneration and neural circuit rewiring. 27 28

However, accumulating evidence indicates that they can also perform beneficial functions. In the early stages of injury, the glial scar may separate the injured site from workable tissue and play significant roles in limiting the spread of the lesion and controlling the scope of inflammatory response. In GFAP ^−/−^ Vim ^−/−^ mice induced cerebral hypoxia-ischemia, a study found that reactive astrocytes are similar in number but lose their hypertrophy and other reactive hyperplasia phenotypes, and the expression of CSPG around the lesion of GFAP ^−/−^ Vim ^−/−^ mice is reduced while the infarct is significantly increased. 29

Additionally, a recent study found that the ablation of reactive astrocytes surrounding cortex infarction significantly increases blood loss and impairs the remodeling of neurovascular units, while vascular structures in non-ischemic brains are not affected by focal astrocyte ablation, 30 showing that reactive astrocytes are a key component in vascular repair and cell medium remodeling after cerebral ischemia.However, the premature inhibition of glial scar formation at the edge of the ischemic core area may cause the spread of damage from the lesion region. 29 31 Consequently, the glial scar in the early stages of ischemia is essential to maintain tissue integrity and reduce further inflammatory damage.

## ASTROCYTE-MEDIATED NEUROGENESIS AFTER ISCHEMIC STROKE

**Figure 2:**
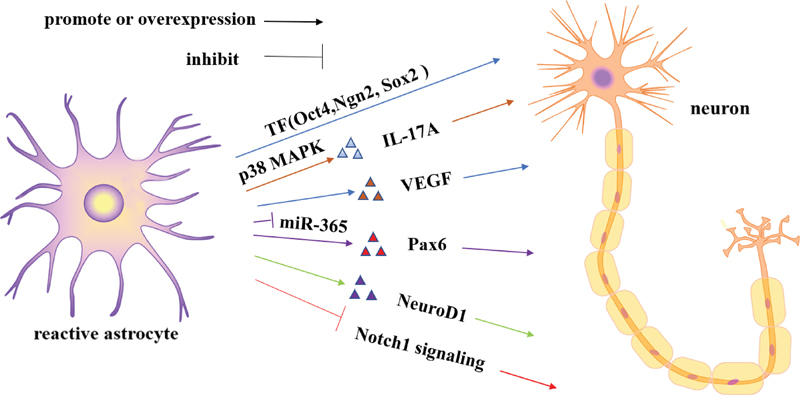
Molecular mechanisms of astrocytes promote neurogenesis in ischemic stroke. In ischemic brain injuries, IL-17A (through p38MAPK signaling pathway) and VEGF secreted by reactive astrocytes promote neurogenesis. Overexpression of transcription factors (Pax6, NeuroD1, Oct4, Sox2, and Ngn2) and inhibition of Notch signaling pathways enhance the transdifferentiation of astrocytes into functional neurons.

Neurogenesis is exceedingly limited in the adult brain. This is true for most mammals, except for the especial astrocytes in the subventricular zone and hippocampal dentate gyrus that have stem cell characteristics and constantly produce new neurons'. However, in response to cerebral ischemia, a growing number of studies have found that astrocytes in the striatum can be transformed into neurons. 32 33 Both in vivo and in vitro studies have shown that astrocytes have the characteristics of stem cells and can differentiate into neurons under certain conditions 34 35 36 37 ( Figure 2 ).

Interestingly, small molecules efficiently reprogram human astrocytes in culture into functional neurons, and human astrocyte-converted neurons can survive in the mouse brain and integrate into the local neural circuits in vivo. 38 Using a model of cerebral ischemia in mice, with cell tracing technology, researchers found that astrocytes are transformed into neurons at the injury site by inhibiting the Notch signaling pathway, which promotes nerve repair. 33

A recent study used single-cell RNA sequencing to analyze the inherent genetic characteristics of astrocytes exposed to middle cerebral artery occlusion (MCAO) conditions, 39 revealing how astrocytes in the brain acquire the ability to form new neurons. This approach uncovers which genes are turned on or off, resulting in an analysis of the gene activity profile for each astrocyte. In Rbpj ^fl/fl^ mice, an infusion of epidermal growth factor can enable stalled striatal astrocytes to initiate the transit amplifying divisions and resume neurogenesis. 39 These results demonstrate that parenchymal astrocytes display neural stem cell properties and that targeted interventions can guide them to complete neuronal differentiation.

Current studies have confirmed that under certain conditions, or by intervening in some signals, such as cytokines, 40 transcription factors, 38 and small molecule compounds, 41 astrocytes have the potential to transdifferentiate or reprogram into neurons. 42 For example, studies have found that astrocytes secrete inflammatory factors that promote stroke recovery. 43

Interleukin-17 A is mainly secreted by astrocytes. When activated, these cells release IL-17A through p38 mitogen-activated protein kinase (MAPK) signaling the pathway to induce neuronal differentiation, while downregulation of IL-17A inhibits neuronal differentiation. 43 Additionally, the vascular endothelial growth factor (VEGF) promotes transdifferentiation of striatal astrocytes into new, mature neurons after ischemic brain injury. 44

Astrocytes can be reactivated in response to ischemic or traumatic brain injury, characterized by increased expression of related proteins. For example, octamer-binding transcription factor 4 (Oct4), self-renewing and pluripotency with neural stem cells (Sox2), and participating in self-renewal of undifferentiated embryonic stem cells (Pax6) 45 can convert astrocytes into neurons. 40

Additionally, neurogenin2 (Ngn2) is an important transcription factor involved in neuronal differentiation, and in the mouse model of traumatic brain injury, it was found that astrocytes can target the formation of neuronal lineage via the overexpression of Ngn2. 46 Remarkably, NeuroD1 is an endogenous neural transcription factor. Recent studies have shown that in rodents or non-human primates with induced cerebral infarction, the NeuroD1-mediated transformation of astrocytes into neurons in situ can regenerate a large number of functional neurons after ischemic brain injury, thereby promoting the recovery of nerve function. 47 48

Astrocytes in glial scars are reprogrammed into functional neurons, 49 showing the therapeutic potential of nerve tissue regeneration after brain injury. Furthermore, the astrocyte-converted neurons not only help to replace lost neurons, but also reduce growth inhibitory factors, creating a more suitable microenvironment for neuronal outgrowth and synaptic integration. Thus, a functional neuronal regeneration of reactive astrocytes provides a potential therapeutic strategy for ischemic stroke.

## ASTROCYTE-MEDIATED PHAGOCYTOSIS AFTER ISCHEMIC STROKE

Until now, phagocytosis has been thought to be limited to specialized phagocytes, such as the microglia in the brain. 50 51 However, there is increasing evidence that nonprofessional phagocytes (e. g. astrocyte) can also take part in this process. 52 53 Large numbers of debris from dying/dead cells can overwhelm the phagocytic capacity of microglia, 54 allowing astrocytes to function as powerful supportive clearance systems.

Recent studies have found that astrocytes have a strong phagocytic ability, and that they participate in the elimination of synapses and axons, 53 55 as well as neuronal fragments in the brain, 56 even under normal conditions. Notably, human astrocytes show phagocytic capacity and strengthen the phagocytic function of microglia in coculture experiments. 57

A genetic analysis study showed that astrocytes are enriched in genes that participate in phagocytic pathways, such as phagocytic receptors, integrins, and opsonins. 58 Both in vivo and in vitro studies have shown that astrocytes engulf synapses through MEGF10 and MERTK (two phagocytic receptors) pathways, and actively promote activity-dependent synaptic elimination. 59

More importantly, a recent study has confirmed for the first time that the astrocyte is an important participant in the elimination of synapses, consistently eliminating excessive adult excitatory synaptic connections in response to neuronal activity, indicating that astrocytes are essential for controlling the number and plasticity of synapses. 53

Wan et al. found the increased lipocalin-2 (LCN2) and low-density lipoprotein receptor-related protein 1 (LRP1) improved astroglial myelin phagocytosis, and Lcn2 ablation or Lrp1 knockdown alleviated demyelination and reversed white matter lesions, suggesting that astrocyte LCN2/LRP1 signaling is required for myelin phagocytosis and subsequent demyelination after focal cerebral ischemia. 60 Therefore, regulating the phagocytosis of astrocytes to restore synaptic connectivity or myelination may be a new therapeutic strategy of ischemic stroke.

Recent studies have shown that the reactive astrocytes induced by ischemia are involved in phagocytosis to clear neuronal debris. 61 Astrocytes can swallow neuronal materials including synapses, apoptotic neurons, and degenerated axons, as well as various toxic proteins. The ATP-binding cassette transporter A1 (ABCA1) and the molecules in its pathway, such as multiple EGF-like-domains 10 (MEGF10) and the engulfment adapter phosphotyrosine binding domain containing 1 (GULP1), are the responsible molecules for phagocytosis.

A recent research reported that reactive astrocyte highly Increases ABCA1 and its related protein, MEGF10, after ischemia. 61 Further studies have found that knockdown or knockout of ABCA1, MEGF10, or GULP1 can significantly reduce the phagocytic capacity of astrocytes and increase cerebral infarct volume, indicating that the removal of neuronal fragments via an ABCA1-MEGF10-GULP1 pathway-mediated phagocytosis of the reactive astrocyte is essential for nerve recovery. However, the phagocytic kinetics of professional and nonprofessional phagocytes are very different, with professional phagocytes having a stronger phagocytic ability. 51 62 Compared with the microglial phagocytosis, the astrocytic phagocytosis starts later but lasts longer. 52 Even in pathological circumstances, astrocytes do not move as fast as microglia, indicating astrocytes may not participate in the acute clearance of injured tissues in the ischemic core area. 63 Therefore, phagocytic astrocytes may promote brain microenvironment remodeling and nerves recovery in the ischemic penumbra.

## REACTIVE ASTROCYTES AND ISCHEMIC TOLERANCE AFTER STROKE

Ischemic preconditioning (IPC) or tolerance is an endogenous neuroprotective mechanism. In this way, a mild ischemic attack can make the brain resistant to subsequent, more severe, ischemic damage.

Currently, most studies on ischemic tolerance or IPC only focus on neuronal cells, but ischemic tolerance can be induced by multistep mechanisms through a variety of cell types, including neurons, astrocytes, and microglia. 64 65 An increasing number of studies have confirmed that astrocytes play a key role in inducing ischemic tolerance and protecting neurons. 66 67

Astrocytes take up glucose via the glucose transporter (GLUT). The expression of this transporter is significantly increased in reactive astrocytes, 68 which produce lactic acid through glycolysis, being controlled by 6-phosphofructo-2-kinase/fructose-2, 6-bisphosphatase-3 (Pfkfb3), 69 or glycogenolysis in ischemic conditions. 70 Lactate can then be transported from the cell by the monocarboxylate transporter 1 or 4 (MCT1/4) and exported into neurons via MCT2. 71 Under ischemic conditions, researchers found an increased flux on lactate production reactions in human astrocytes via the genome-scale reconstruction. 72

The IPC in astrocytes transfers ischemic tolerance to neurons, and the underlying factor that mediates this protective effect is the soluble transport of lactic acid. 73 Studies have found that after ischemic preconditioning, astrocytes increase cell survival rate by up-regulation of GLUT1 and GLUT3. 74 75 The nuclear erythroid 2-related factor 2 (Nrf2) is an antioxidant transcription factor, and IPC protects astrocytes against oxygen glucose deprivation by the Nrf2 pathway. 76 In the OGD-induced astrocyte injury model, studies have confirmed that ischemic preconditioning protects astrocytes from ischemic damage by inducing 14-3-3γ (a multifunctional scaffolding protein) expression and maintaining energy metabolism in a variety of ways. 77

Additionally, astrocytes are sensitive to environmental changes and can be affected by even minor injuries, such as transient ischemia or IPC. 78 79 Astrocytes exert neuroprotective effects by releasing neurotransmitters such as ATP and glutamate, 80 81 which, in turn, act on transducers of ischemic tolerance to provide neuroprotection against succeeding severe injure. 82 The glial glutamate transporter-1 (GLT-1) is primarily distributed in astrocytes and is responsible for 90% of glutamate uptake. Many studies have showed that GLT-1 upregulation plays a vital role in inducing ischemic tolerance by preventing excessive glutamate accumulation and terminating multiple downstream death-signaling-cascades. 83 84 Compared with normal astrocytes, activated astrocytes in the ischemic area are closely related to the spatiotemporal pattern of ischemic tolerance after IPC. 65 85

The P2X7 receptor, as an ion channel forming ATP receptor, is selectively upregulated in activated astrocytes. Studies have shown that the P2X7 receptor is required for ischemic tolerance. Further research found that hypoxia-inducible-factor-1a (HIF-1a) is upregulated by IPC in a P2X7 receptor-dependent manner. The increase of HIF-1a is persistent in astrocytes, and the receptor also exhibits a slow and enduring expression. 81 This time difference of the P2X7 receptor may allow HIF-1a of astrocytes to induce ischemic tolerance, which, in turn, can produce many neuroprotective molecules, such as erythropoietin and vascular endothelial growth factor. 85 86 Thus, astroglia-mediated ischemic tolerance provides a powerful and lasting neuroprotective effect against ischemic injuries.

## SUMMARY AND PROSPECTS

**Figure 3:**
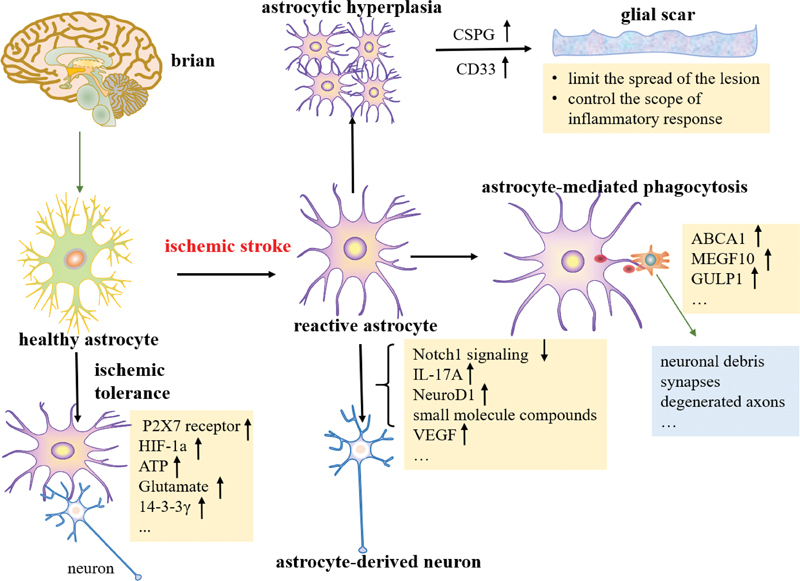
The neuroprotective mechanisms of astrocytes in reactive astrocytosis, neurogenesis, phagocytosis, and ischemic tolerance after ischemic stroke.

Astrocytes are the most abundant cell type in the central nervous system and play a vital role in maintaining normal brain function. This review summarizes the role of reactive astrocyte hyperplasia and glial scars in ischemic strokes, as well as the recent advances in the neuroprotective regulations of reactive astrocyte-mediated neurogenesis, phagocytosis, and ischemic tolerance after cerebral ischemia ( Figure 3 ). However, the regulatory mechanism of astrocytes is not yet fully understood. Moreover, because of the limited access to human brain tissue and technical limitations, there's particularly little study on human astrocytes induced by ischemia.

As a matter of fact, reactive astrocytes have neuroprotective or neurotoxic effects, and can regulate the astrocytes to promote the recovery of brain injury and nerve function, which brings new directions and challenges to therapeutic approaches for ischemic stroke.

In conclusion, the evidence of astrocyte-mediated neuronal transformation, phagocytosis and ischemic tolerance opens a novel perspective for the treatment of ischemic strokes. After cerebral ischemia, reactive astrocytes are highly plastic and heterogeneous in terms of morphology, proliferation, and gene expression. Therefore, further studies on the dynamics of reactive astrocytes at the molecular and cellular levels will provide new therapeutic strategies of ischemic stroke.
